# Developing and piloting a communication assessment tool assessing patient perspectives on communication with pharmacists (CAT-Pharm)

**DOI:** 10.1007/s11096-022-01382-y

**Published:** 2022-02-24

**Authors:** Daniela Scala, Sara Mucherino, Francesca Wirth, Valentina Orlando, Piera Polidori, Maria Ernestina Faggiano, Daniela Iovine, Paola Saturnino, Francesco Cattel, Alberto Costantini, Corrado Giua, Gregory Makoul, Lilian M. Azzopardi, Enrica Menditto

**Affiliations:** 1 Nuclear Medicine, AORN “A. Cardarelli”, Naples, Italy; 2 CIRFF, Center of Pharmacoeconomics and Drug Utilization Research, Department of Pharmacy, University of Naples Federico II, Naples, Italy; 3 Department of Pharmacy, Faculty of Medicine and Surgery, University of Malta, Msida, Malta; 4 Department of Clinical Pharmacy, ISMETT, Palermo, Italy; 5 Pharmacy Department, AOU Policlinico Di Bari, Bari, Italy; 6 Pharmacy Department, AORN “A. Cardarelli”, Naples, Italy; 7 Farmacia Ospedaliera A.O.U. Città Della Salute E Della Scienza Di Torino, Turin, Italy; 8 U.O.C. Farmacia Ospedaliera Aziendale Ospedale “Santo Spirito” ASL Pescara, Pescara, Italy; 9 Società Italiana Farmacia Clinica (SIFAC), Cagliari, Italy; 10 PatientWisdom, Inc., Madison CT USA and Yale School of Medicine, New Haven, CT USA

**Keywords:** Communication, Communication assessment tool, Community pharmacy, Hospital pharmacy, Patient-pharmacist relationship, Patient empowerment

## Abstract

**Background:**

Effective communication strategies in health care help to enhance patient empowerment and improve clinical outcomes.

**Objective:**

Adapt the original Communication Assessment (CAT) instrument for the pharmacist profession (CAT-Pharm) and to test its validity and reliability in two different settings.

**Setting:**

Five hospital pharmacies in Italy and five community pharmacies in Malta.

**Method:**

Pilot study involving a standardized multi-step process adhering to internationally accepted and recommended guidelines. Corrections and adjustments to the translation addressed linguistic factors and cultural components. CAT-Pharm, compared to the original CAT, maintained 10 out of the 14 items: one was slightly modified; three were changed to better fit the pharmacist role; one was added.

**Main outcome measures:**

CAT-Pharm development and testing its practicality to assess patient perceptions of pharmacists’ interpersonal and communication skills.

**Results:**

CAT-Pharm was tested on 97 patients in the Italian setting and 150 patients in the Maltese setting to assess the practicality of the tool and its usefulness in investigating gaps and priorities for improving pharmacist-patient communication. Results Show reliability and internal validity of the CAT-Pharm tool. The analysis of patient perceptions of communication with the pharmacist in Italy indicated differences from that in Malta. The different settings provided insight into the utility of CAT-Pharm.

**Conclusion:**

This study provided a valid and reliable tool that could be applied to assess patient perception of the pharmacist's communication abilities.

**Supplementary Information:**

The online version contains supplementary material available at 10.1007/s11096-022-01382-y.

## Impact Statements

- Promoting strategies of communication in the hospital and community pharmacy setting is an essential element to improve patients’ empowerment in the interest of patient safety and quality of care.
- Pharmacists are in an ideal position to facilitate communication between physicians and patients since they have frequent contacts with patients and extensive knowledge about drug therapy, reason why communication tools should be feasible tools to be implemented in hospital and community pharmacy settings.
- Ad-hoc validated communication tools can help pharmacists to reflect on their interpersonal and communication skills with the goal of reinforcing strengths and identifying areas that would require more attention to improve patient empowerment.

## Introduction

Communication between health professionals and patients is a key element contributing to patient safety and quality care. Patient evaluation of the communication skills of health professionals can have a profound effect on perceptions of quality of treatment received and may influence patient satisfaction and behavioural intentions [1, 2].

There is evidence that effective communication can generate a degree of trust and improve patient empowerment, resulting in better clinical outcomes of chronic medical issues, such as diabetes, hypertension, obesity, HIV/AIDS, cancer, cardiovascular disease, and rheumatoid arthritis [3–6]. Promoting strategies of communication in the health system is an essential element for preventing errors and failures in health care [7].

Within this context, the role of “communicator” is one of the essential functions attributed to pharmacists by the World Health Organization (WHO) [8]. The WHO proposed the concept of the “Seven-star pharmacist” in 1997, which evolved and was taken up by the International Pharmaceutical Federation and covered the following roles: Caregiver, decision-maker, communicator, manager, life-long learner, teacher, and leader [9, 10].

Pharmacists are in an ideal position to facilitate communication between physicians and patients since they have frequent contact with patients, have extensive knowledge about drug therapy, and are equipped to provide information, monitor patients’ experiences and adherence, and co-ordinate care between different healthcare professionals [2, 11, 12]. Pharmacists’ contribution is related to supporting patients in safe and effective medicines use, whether the pharmacist is practicing in a community or hospital setting. In both cases the pharmacist contributes to ensuring access to medicinal products and patient consultation.. Tools to assess patient perceptions of pharmacists’ interpersonal and communication skills are considered to be useful in supporting development of this professional skill. In 2007, Makoul et al. developed the Communication Assessment Tool (CAT) aimed to help physicians to reflect on their interpersonal and communication skills with the goal of reinforcing strengths and identifying areas that require more attention for improvement [13]. The CAT has been translated and cross-culturally adapted to many languages, including Italian [13–15].

Although the CAT is a validated tool intended to evaluate communication across different specialties and environments, there is no evidence of specific evaluation of pharmacists’ communication skills.

The aim of this study were to adapt the original CAT instrument to the pharmacist profession (*CAT-Pharm*) and to test its validity and reliability in two different settings.

### Ethics approval

The study was supported by the Italian Society of Hospital Pharmacy (SIFO). Ethics approval was obtained from the Ethics Committee of Cardarelli Hospital in Naples Italy (424/2017). This research was in conformity with the University of Malta's Research Code of Practice and Research Ethics Review Procedures.

## Method

This was a pilot study carried out from June to December 2017 in Italy and from January to June 2018 in Malta. Twelve Italian hospital pharmacists selected from five Centers in the South, Center and North of Italy, and five community pharmacists selected by convenience sampling from each of the five districts of the island of Malta, were involved in this study. The enrolled pharmacists were responsible to administer the questionnaire to the volunteer patients. A reference pharmacist for Malta and one for Italy assumed responsibility for the final collection of all paper questionnaires.

### Adaptation of CAT to pharmacist profession

An International working group (an instrument developer, pharmacists from English speaking countries, researchers with expertise in statistics and in patient reported outcomes, and pharmacists fluent in English with Italian as their native language) helped in development of the tool, adaptation and validation analyses of the instrument, and translation into English. This group consisted of A final modified version of CAT Tool, the CAT-Pharm, was obtained, and this version was translated into English and Maltese. The final version includes additional elements designed to collect self-reported demographic information (age, ethnicity, gender).

**Fig. 1:**
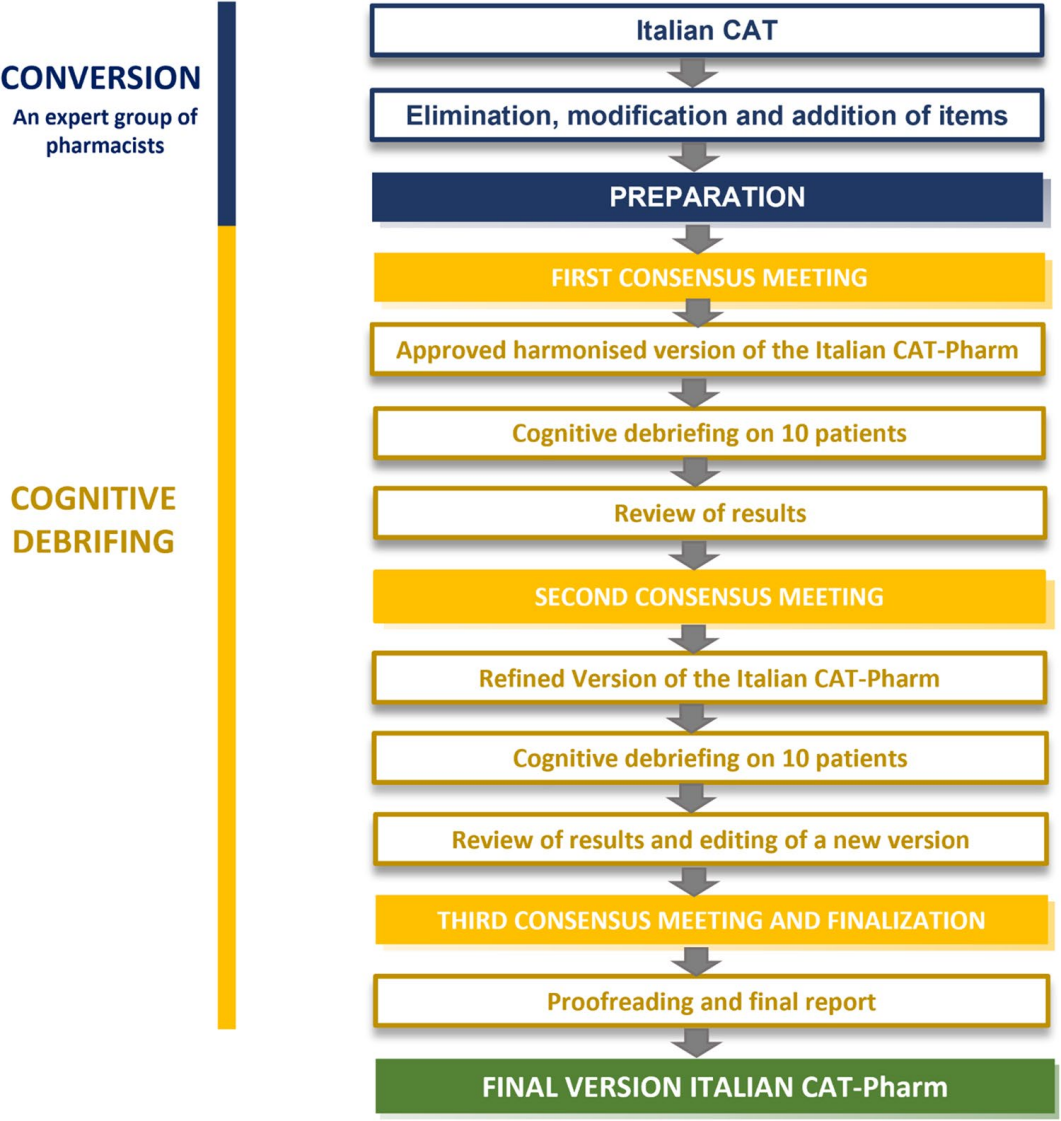
Process flow chart for obtaining Italian CAT-Pharm

As shown in Fig. 1, adaption to the pharmacist profession was achieved through five steps:

- *Step 1*: An expert group composed of 4 Italian pharmacists reviewed the CAT giving suggestions about elimination, modification, addition of items.
- *Step 2*: Consensus meeting to reach a *harmonized version of the Italian CAT-Pharm* that includes 15 items which measure patient perceptions of pharmacist communication, all measured on a 5-point response scale (1=poor; 2=fair; 3=good; 4=very good; 5=excellent). Compared to the original CAT, the harmonized version of the Italian CAT-Pharm consists of an additional item. Minor changes to the instructions were incorporated.
- *Step 3*: Cognitive debriefing on 10 patients to assess if the questionnaire is easy to understand. Respondents were administered the *harmonized version of the Italian CAT-Pharm* and were systematically asked to identify what they think each question is asking, whether they can repeat the question in their own words, and what comes to mind when they hear a particular phrase or term. The patients were asked to explain how they selected their answer.
- *Step 4:* Consensus meeting to reach a *refined version of the Italian CAT-Pharm* based on the analysis and discussion of information about comprehension of items and use of the tool in Step 3.
- *Step 5:* The refined version was administered to an additional 10 patients in the same way as the previous version (step 3). Suggestions and comments expressed by respondents were collected and analyzed, yielding a *final version Italian CAT-Pharm* (Supplementary File 1).

Information related to the process of CAT adaptation to the pharmacist profession (CAT-Pharm) are shown in Supplementary Files 2 and 3.

Subsequently, the following two steps were followed to obtain CAT-Pharm in English and Maltese.

- *Step 6*: The final version of Italian CAT-Pharm was translated into English by an Italian mother tongue fluent in English (forward translation) and the following back translation was done by an English mother tongue. After a back translation review, a cognitive debriefing was done by three pharmacists and three laypersons. Final stages included proofreading and finalization of the English version (Supplementary file 4). The entire process of language adaptation and translation was carried out according to internationally accepted and recommended guidelines of International Society of Pharmacoeconomics and Outcome Research (ISPOR) and recommendations made by the WHO about the process of translation and adaptation of instruments [16–18].
- *Step 7:* The English version of CAT-Pharm was translated to Maltese language by a Maltese linguist, back translated to English by an English mother tongue, and both versions were validated by two pharmacists and three laypersons. The process of language adaptation and translation was carried out according to the same guidelines [16–18]. Applicability testing of CAT-Pharm in English and Maltese was carried out in one community pharmacy with 10 patients.

**Fig. 2:**
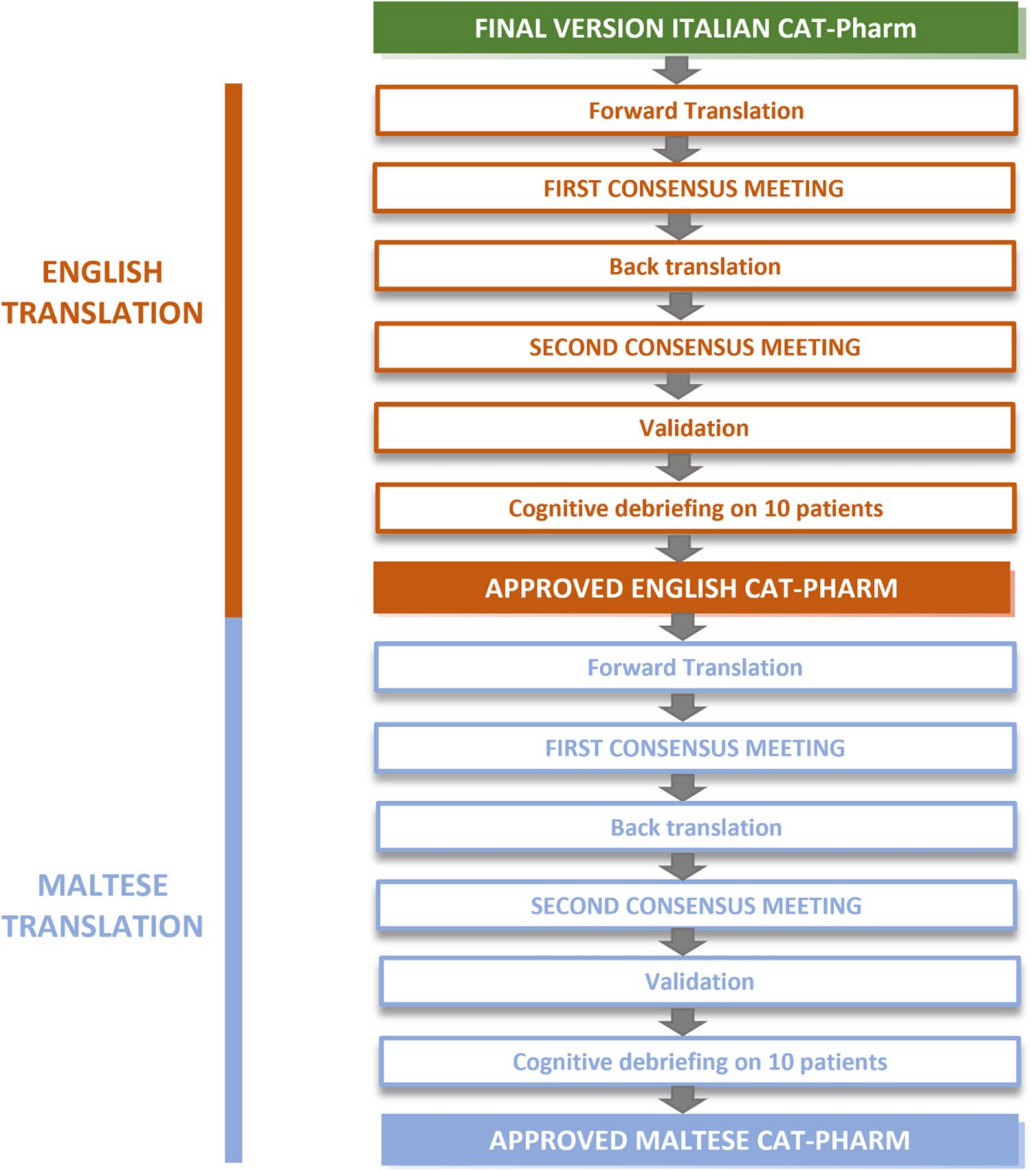
Process flow chart for obtaining the English and Maltese CAT-Pharm

These two steps are graphically represented in Fig. 2.

### Setting, participants and eligibility criteria

CAT-Pharm was tested in Italy and Malta. In Italy, the setting was the hospital where pharmacists are involved in ensuring access to medicinal products, a consulting relationship with the patient and act as part of the multidisciplinary team. CAT-Pharm in Italy was applied to 97 outpatients recruited by convenience sampling in each of the five hospital pharmacies involved in the study. Patients interviewed and engaged by Italian hospital pharmacists were not inpatients, hence, patients coming to the pharmacy after a visit and with a drug prescription were invited to fill the CAT-Pharm. CAT-Pharm in Malta was applied to 30 patients recruited by convenience sampling in each of the five community pharmacies (N = 150). In each community pharmacy, 30 patients presenting a prescription to the same pharmacist, were handed the CAT-Pharm and invited to complete either the English or Maltese version. This process provided the opportunity to test the utility of applying the CAT-Pharm tool in community pharmacy setting.

In both Italian and Maltese settings, the pharmacist submitted the questionnaire to the volunteer patients. Anonymity of the completion of the tool was ensured. One day was dedicated to data collection per pharmacy.

### Statistical analyses

Validity (internal, external) and reliability assessments were required to determine the psychometric properties of the developed CAT-Pharm instrument. To investigate t validity of each item of the pharmacist-adapted CAT tool, a confirmatory factor analysis was performed. Sample adequacy was measured by Kaiser–Meyer–Olkin (KMO) and Bartlett's sphericity test. To confirm factor structure, a Oblimin direct rotation with Kaiser normalization was performed. Correlations between items were assessed using the Pearson's correlation test. The Chi-square test was used to compare the proportion of patients who rated a given item ‘Excellent’ between the two settings. A *p*-value < 0.05 was considered statistically significant. As questionnaire responses were structured with a 5-point Likert scale (poor; fair; good; very good; excellent), Cronbach’s alpha internal consistency reliability was performed to assess internal consistency for the translated CAT overall score. As in the original scale development, psychometric analysis indicated that’Excellent’ maps onto ‘Yes’, and all the other response options (i.e. poor; fair; good; very good) map onto “No” [13]. Accordingly, and consistent with previous use of the CAT, results are presented as the percentage of participants who provided ratings of ‘Excellent’. Percentage of ‘Excellent’ responses was calculated from the total number of respondents to the individual question. Analyses were performed using SPSS Statistics for Windows, version 17.1 (SPSS Inc.Released 2008. Chicago, IL; USA).

## Results

### Developed CAT-Pharm tool

The final version of CAT-Pharm was obtained by making minor changes to the original CAT (Table 1). References in the original CAT to ‘‘your doctor’’ or ‘‘the doctor’’ were changed to ‘‘your pharmacist’’ or ‘‘the pharmacist’’, and reference in the original CAT to “health” were changed to “prescribed therapy” (item 3).

**Table 1:** Cross-cultural adaptation and translation of the CAT-Pharm

Item	Original items	Adaptation for the pharmacists’ profession	Changes compared to original CAT
Item 1	Greeted me in a way that made me feel comfortable	Greeted me in a way that made me feel comfortable	-
Item 2	Treated me with respect	Treated me with respect	-
Item 3	Showed interest in my ideas about my health	Showed interest in my ideas about the prescribed therapy	**Minor changes**
Item 4	Understood my main health concerns	Understood my main health concerns	-
Item 5	Paid attention to me (looked at me, listened carefully)	Explained how to correctly follow the prescribed therapy	**Changed**
Item 6	Let me talk without interruptions	Let me talk without interruptions	-
Item 7	Gave me as much information as I wanted	Gave me as much information as I wanted	-
Item 8	Talked in terms I could understand	Talked in terms I could understand	-
Item 9	Checked to be sure I understood everything	Checked to be sure I understood everything	-
Item 10	Encouraged me to ask questions	Encouraged me to ask questions	-
Item 11	Involved me in decisions as much as I wanted	Discussed how to manage any side effects of the prescribed therapy	**Changed**
Item 12	Discussed next steps, including any follow-up plans	Discussed next steps, including any follow- up plans	-
Item 13	Showed care and concern	Asked about my ability to follow the prescribed therapy	**Changed**
Item 14	Spent the right amount of time with me	Spent the right amount of time with me	-
Item 15	–	Discussed possible interactions of the prescribed therapy with other drugs or foods	**Added**

Copyright © Gregory Makoul, PhD MS -- All rights reserved

Item 5 “Paid attention to me (looked at me, listened carefully)” was changed to “Explained how to correctly follow the prescribed therapy” and item 11 “Involved me in decisions as much as I wanted” was changed to “Discussed how to manage any side-effects of the prescribed therapy”. Item 13 “Showed care and concern” was changed to “Asked about my ability to follow the prescribed therapy” and an additional item (item 15) was added “Discussed possible interactions of the prescribed therapy with other drugs or foods”.

Validity of the CAT-Pharm items was assessed. Pearson’s correlation test showed significant positive correlations between CAT-Pharm items. The correlation coefficients ranged from 0.26 to 0.86.

The results of the Bartlett’s test of sphericity were KMO = 0.92 and χ2 = 2969.34 (df = 105, *p* < 0.01), indicating that the correlation matrix was suitable for factor analysis. A two-factor solution was found identifying two questionnaires macro-areas. Factors 1 (the first six items) is focused on the confidential and familiar relationship pharmacist-patient. Factor 2 (items 7–15) is focused on investigating the correct activity of the pharmacist towards the patient. Results of confirmatory factor analysis are showed in the Supplementary File 5.

Reliability results indicated very high overall scale reliability for the 15 CAT-Pharm items (Cronbach’s alpha = 0.95).

### Applicability of the tool

The CAT-Pharm was tested on 97 patients in the Italian setting and 150 patients in the Maltese setting.

In the Italian setting, 51 patients (52.6%) were between 45 and 64 years of age, 50 participants (51.5%) were male and 90 (92%) were native Italian speakers. In Malta, 63 patients (42.0%) were between 65 and 84 years of age, 89 participants (59.3%) were female and 146 (97.3%) were Caucasian. In the Maltese setting 147 patients (98%) filled the questionnaire in English. Demographic characteristics of the two populations are shown in Table 2.

**Table 2:** Demographic characteristics of patients completing CAT-Pharm

Demographic characteristics	Italy		Malta	
	N = 97		N = 150	
	n	%	n	%
Gender				
Male	50	51.5	61	40.7
Female	47	48.5	89	59.3
Age in years				
≤ 24	1	1.0	2	1.3
25–44	18	18.6	48	32.0
45–64	51	52.6	36	24.0
65–84	25	25.8	63	42.0
≥ 85	–	–	1	0.7
Nationality/Ethnicity				
Native Italian speaker	90	92.0	–	–
Non-native Italian speaker	7	7.2	–	–
Caucasian	–	–	146	97.3
Hispanic or Latino	–	–	3	2.0
Asian	–	–	1	0.7
Language in which CAT-Pharm was completed				
Italian	97	100	–	–
English	–	–	147	98.0
Maltese	–	–	3	2.0
Had the patient seen the pharmacist before?				
No	65	67.0	10	6.7
Yes, but only once	19	19.6	16	10.7
Yes, more than once	13	13.4	124	82.7

Table 3 shows differences in ‘Excellent’ rating scores for each CAT-Pharm item in the two settings. The ‘Excellent’ scores of Italian CAT-Pharm items ranged from 12.4% to 55.7%. The highest-scoring items were ‘‘Talked in terms I could understand’’ (55.7%) and ‘‘Treated me with respect’’ and “Spent the right amount of time with me” (both 53.6%). The lowest-scoring item was ‘‘Discussed next steps, including any follow-up plans’’ (12.4%).

**Table 3:** Percentage of excellent ratings for individual CAT-Pharm items

Item	Statement	Excellent ratings (%)		*Chi-square Test (P)Value*
		Italy	Malta	
		N = 97	N = 150	
Item 1	Greeted me in a way that made me feel comfortable	49.5	76.0	< *0.001*
Item 2	Treated me with respect	53.6	86.0	< *0.001*
Item 3	Showed interest in my ideas about the prescribed therapy	42.3	64.7	*0.001*
Item 4	Understood my main health concerns	36.1	67.3	< *0.001*
Item 5	Explained how to correctly follow the prescribed therapy	30.9	86.0	< *0.001*
Item 6	Let me talk without interruptions	45.4	68.0	*0.001*
Item 7	Gave me as much information as I wanted	38.1	81.3	< *0.001*
Item 8	Talked in terms I could understand	55.7	88.0	< *0.001*
Item 9	Checked to be sure I understood everything	48.5	65.3	*0.014*
Item 10	Encouraged me to ask questions	25.8	46.7	*0.001*
Item 11	Discussed how to manage any side effects of the prescribed therapy	26.8	60.7	< *0.001*
Item 12	Discussed next steps, including any follow-up plans	12.4	47.3	< *0.001*
Item 13	Asked about my ability to follow the prescribed therapy	32.0	60.7	< *0.001*
Item 14	Spent the right amount of time with me	53.6	75.3	*0.001*
Item 15	Discussed possible interactions of the prescribed therapy with other drugs or foods	21.6	63.3	< *0.001*

The ‘Excellent’ scores obtained from the Maltese setting using the English and Maltese versions of the tool ranged from 46.7% to 88%. The highest-scoring items were ‘‘Talked in terms I could understand” (88%) and “Treated me with respect”and “Explained how to correctly follow the prescribed therapy” (both 86%). The lowest-scoring item was ‘‘Encouraged me to ask questions’’ (46.7%). A statistically significant difference in response between the Italian and Maltese setting was detected for all the items. Higher ratings were observed from the Maltese setting (Table 3).

## Discussion

The uniqueness of this study is that it presents a new tool to be used by patients to rate the communication with pharmacists related to prescribed medications. Items in the CAT and CAT-Pharm have the same communication tasks. The CAT-Pharm, compared to the original CAT, maintained 10 out of the 14 items, one item was slightly modified, three items have undergone changes to reflect the contribution of the pharmacist and one item was added to discuss possible interactions between prescribed therapy and other drugs or food. Although the CAT-Pharm is proposed as an assessment tool specific for pharmacist-patient relationship to reflect on their interpersonal and communication skills, the original purpose of the CAT-tool developed by Makoul et al. in 2007 [13], was maintained and this was confirmed by the results of the factor analysis. The first six items are aimed at investigating the confidential relationship between the patient and the pharmacist and how comfortable the patient feels with the pharmacist. The remaining items are more focused on investigating the correct activity of the pharmacist towards the patient, i.e. including the patient in decisions, discussing next steps.

Given the usefulness of the tool specifically directed at the pharmacist-patient relationship, its applicability to all settings and contexts cannot be taken for granted. Relying on validated guidelines is crucial when carrying out modifications to psychometric questionnaires for adaptation to different professional groups or a different setting. CAT-Pharm reliability was confirmed by Cronbach’s alpha values, validity confirmed by factor analysis, and internal validity assessed by administering and evaluating responses from a small sample of patients from the two different settings.

CAT-Pharm external validity should be evaluated for the application to other settings which will require cross-cultural validation prior to implementation. Implementation of CAT-Pharm tool may be suggested as a method to assess patients' views of pharmacists' communication behavior and to identify areas that require more attention for improvement as part of professional development programs or as a competency development measurement tool for pharmacy students.

It is interesting to note that analysis of patient perceptions of communication with the pharmacist in Italy demonstrated differences from that in Malta. Usually, the community pharmacist has more frequent and direct contact with patients compared to the hospital pharmacist, which explains why 67% of the patients who participated in Italy said they had never seen the pharmacist before, while in Malta only 6.7% of Maltese patients stated this. The largest difference was observed in the response to the question "Discussed next steps, including any follow-up plans" underlining how the community pharmacist has a continuous and frequent interaction with the same patient. Particularly, few patients in the Italian context rated as ‘Excellent’ the attitude of the pharmacist in discussing possible interactions of prescribed therapy with other drugs and food or the management of possible side-effects.

Notably, other significant differences were observed in patient perceptions of pharmacist communication methods, which were always greater in the Maltese community setting. It is to be understood that a high patient regard of community pharmacists’ services including clinical services related to medication management has been reported for community pharmacy practice in Malta [19, 20]. This is explained through the highly evolved patient-centered curriculum adopted in pharmacy education in Malta [21]. It is noteworthy that assessing the difference between Italy and Malta was not among the primary objectives of the study; however, significant differences emerged that warrant further investigation in a larger cohort of patients. The utility of the tool to detect differences in practice is an application of the tool to be investigated in terms of its use as a performance indicator for service development within pharmaceutical health systems [22–24].

Confirming results of previous studies, patients desire more opportunities to ask questions and for more active involvement in decisions regarding their care [13, 25–27]. The clinical relationship must serves to obtain information from the patient to identify their needs and understanding of the care plan as well as to provide the opportunity to patients to share their thoughts and questions. [28, 29].

### Limitations

A limitation of this study is the nature of assessment of validity where the tool was measuring communication with the pharmacist and seeking response by the participants availing themselves of the service to comment on the service received. Other limitations of the study included the small sample size and the adoption of expert group from two countries rather than a Delphi technique.

A limitation is that the study looked at content validity and did not assess construct validity. The high Cronbach’s alpha values may indicate a redundancy of some items in the CAT-Pharm tool. In this study, the aim was to adapt the original CAT tool to the pharmacist profession and therefore the potential redundancy of items was not addressed in this paper. In further studies, the redundancy may be considered prior to undertaking construct validity and external validity. The next step will be to perform the study on a larger sample for external validity analyses and to ensure generalizability of the tool. During the external validation phase ethnicity questions will be added to all versions of the tool- together with the possibility of presenting the questionnaire in English to all patients.

## Conclusion

This pilot study demonstrated that the developed CAT-Pharm tool may be applied to different pharmacy settings and is a valid and reliable tool that could be submitted for further psychometric testing to evaluate its contribution as an instrument to assess patient perception of the pharmacist's communication abilities. CAT-Pharm has the potential to be useful for pharmacists to reflect on their interpersonal and communication skills with the ideal goal of reinforcing strengths and identifying areas that would require more attention to improve patient empowerment.

## Supplementary Information

Below is the link to the electronic supplementary material.

Supplementary file1 (DOCX 53 kb)

Supplementary file2 (DOCX 18 kb)

Supplementary file3 (DOCX 15 kb)

Supplementary file4 (DOC 115 kb)

Supplementary file5 (DOCX 35 kb)
