# Supplementary material for: Developing and piloting a communication assessment tool assessing patient perspectives on communication with pharmacists (CAT-Pharm)
Source: Int J Clin Pharm. 2022 Feb 24;44(4):1037–45. doi: 10.1007/s11096-022-01382-y (PMC9393125; doi:10.1007/s11096-022-01382-y)
Supplement: Supplementary file 1 — Supplementary file1 (DOCX 53 kb) [file 11096_2022_1382_MOESM1_ESM.docx]

**Nome del Farmacista:**

**Strumento di Valutazione della Comunicazione**

La comunicazione con i pazienti è una componente molto importante della qualità dell’assistenza sanitaria. Gradiremmo conoscere le sue impressioni sul modo con cui il suo farmacista comunica con lei. **Le sue risposte sono del tutto confidenziali, per cui Le saremo grati se sarà il più possibile sincero ed obiettivo**.

La sua partecipazione è volontaria e non influirà in alcun modo sull’assistenza sanitaria.

Per favore dia un punteggio al modo di comunicare del suo farmacista.

Segni con una X la sua risposta per ciascuna domanda mostrata di seguito.

Grazie molto

| **Il Farmacista…** | **Scarso** | **Sufficiente** | **Buono** | **Molto Buono** | **Eccellente** |
| --- | --- | --- | --- | --- | --- |
| 1. Mi ha accolto in un modo che mi ha fatto sentire a mio agio | 1 | 2 | 3 | 4 | 5 |
| 1. Mi ha trattato con rispetto | 1 | 2 | 3 | 4 | 5 |
| 1. Ha mostrato interesse per le mie idee sulla terapia prescritta | 1 | 2 | 3 | 4 | 5 |
| 1. Ha capito le mie principali preoccupazioni di salute | 1 | 2 | 3 | 4 | 5 |
| 1. Mi ha spiegato come seguire correttamente lo schema terapeutico prescritto dal medico | 1 | 2 | 3 | 4 | 5 |
| 1. Mi ha lasciato parlare senza interrompermi | 1 | 2 | 3 | 4 | 5 |
| 1. Mi ha fornito tutte le informazioni che volevo | 1 | 2 | 3 | 4 | 5 |
| 1. Ha verificato che avessi capito ogni cosa | 1 | 2 | 3 | 4 | 5 |
| 1. Ha parlato con parole per me facili da capire | 1 | 2 | 3 | 4 | 5 |
| 1. Mi ha incoraggiato a fare domande | 1 | 2 | 3 | 4 | 5 |
| 1. Ha discusso con me come gestire gli eventuali effetti indesiderati provocati dalla terapia | 1 | 2 | 3 | 4 | 5 |
| 1. Ha discusso degli interventi futuri incluso eventuali esami e visite di controllo | 1 | 2 | 3 | 4 | 5 |
| 1. Mi ha chiesto se ero in grado di seguire correttamente lo schema terapeutico prescritto dal medico | 1 | 2 | 3 | 4 | 5 |
| 1. Mi ha dedicato il giusto tempo | 1 | 2 | 3 | 4 | 5 |
| 1. Ha discusso con me delle possibili interazioni della terapia prescritta con altri farmaci e alimenti | 1 | 2 | 3 | 4 | 5 |

**~ continua sull’altro lato ~**

Commenti:

********************************************************************************

Queste informazioni servono per scopi statistici e rimarranno anonime. Per favore segni una sola risposta per ogni domanda.

1. Età ____

1. Sesso _1_ Maschio

_2_ Femmina

1. Ha mai avuto contatti con questo farmacista prima?  _1_ No

_2_ Si, ma solo una volta

_3_ Si, più di una volta

1. Nazionalità:

_1_ Italiana

_2_ Non Italiana

1. Oggi era lei il paziente? _1_ Si

_2_ No, ho accompagnato il paziente

**Grazie**
