# Supplementary material for: Developing and piloting a communication assessment tool assessing patient perspectives on communication with pharmacists (CAT-Pharm)
Source: Int J Clin Pharm. 2022 Feb 24;44(4):1037–45. doi: 10.1007/s11096-022-01382-y (PMC9393125; doi:10.1007/s11096-022-01382-y)
Supplement: Supplementary file 2 — Supplementary file2 (DOCX 18 kb) [file 11096_2022_1382_MOESM2_ESM.docx]

**Supplementary table 2.** Short description of modifications from CAT to CAT-Pharm – Italian version

| **Section/Item** | **Harmonized Italian Version** | | **Refined Italian Version** | | **Final Italian version** | |  |
| --- | --- | --- | --- | --- | --- | --- | --- |
| **Title** | **Discussion** | **Consensus** | **Discussion** | **Consensus** | **Discussion** | **Consensus** |  |
| Items’ numeration was changed in adapting the CAT to pharmacists’ context according to a logical temporal sequence | | | | | | |  |
| **Instruction** | Phisician | *Changed to*  Pharmacist |  |  |  |  |  |
| **CAT Item 3** | “Ha mostrato interesse per le mie idee sulla mia salute” | *Changed to*  “Ha mostrato interesse per le mie idee sulla terapia prescritta” |  |  |  |  |  |
| **CAT Item 5** | “Mi ha prestato attenzione (mi ha guardato, mi ha ascoltato con attenzione)” | *Eliminated* |  |  |  |  |  |
| **CAT Item 11** | “Mi ha coinvolto nelle decisioni sulla mia salute nella misura da me desiderata” | *Eliminated* |  |  |  |  |  |
| **CAT Item 13** | “Ha mostrato attenzione e interesse” | *Eliminated* |  |  |  |  |  |
| **CAT-Pharm**  **Item 5** |  | *New*  “Mi ha dato informazioni su come seguire la terapia prescritta dal medico” | “Mi ha dato informazioni su come seguire la terapia prescritta dal medico” | *Changed to*  “Mi ha spiegato come seguire correttamente lo schema terapeutico prescritto dal medico” |  |  |  |
| **CAT-Pharm**  **Item 13** |  | *New*  “Mi ha chiesto se ero in grado di seguire correttamente la terapia prescritta dal medico” | “Mi ha chiesto se ero in grado di seguire correttamente la terapia prescritta dal medico” | *Changed to*  “Mi ha chiesto se ero in grado di seguire correttamente lo schema terapeutico prescritto dal medico” |  |  |  |
| **CAT-Pharm**  **Item 11** | |  | *New*  “Ha discusso con me come gestire gli eventuali effetti collaterali provocati dalla terapia” |  |  | “Ha discusso con me come gestire gli eventuali effetti collaterali provocati dalla terapia” | *Changed to*  “Ha discusso con me come gestire gli eventuali effetti indesiderati provocati dalla terapia” |
| **CAT-Pharm**  **Item 12** | |  |  | “Ha discusso sulle prossime cose da fare, incluso eventuali programmi di esami e visite di controllo” | *Changed to*  “Ha discusso degli interventi futuri, incluso eventuali programmi di esami e visite di controllo” |  |  |
| **CAT-Pharm**  **Item 15** | |  | *New*  “Ha discusso con me delle possibili interazioni della terapia prescritta con altri farmaci e alimenti” |  |  |  |  |
