# Supplementary material for: Developing and piloting a communication assessment tool assessing patient perspectives on communication with pharmacists (CAT-Pharm)
Source: Int J Clin Pharm. 2022 Feb 24;44(4):1037–45. doi: 10.1007/s11096-022-01382-y (PMC9393125; doi:10.1007/s11096-022-01382-y)
Supplement: Supplementary file 3 — Supplementary file3 (DOCX 15 kb) [file 11096_2022_1382_MOESM3_ESM.docx]

**Supplementary table 3.** Short description of modifications – English version

| **Section/Item** | **Reconciled English Version** | | **Back Translation Review** | | **Harmonized English Version** | | **Refined English Version** | | **Final English version** | |  |  |
| --- | --- | --- | --- | --- | --- | --- | --- | --- | --- | --- | --- | --- |
| **Title** | **Discussion** | **Consensus** | **Discussion** | **Consensus** | **Discussion** | **Consensus** | **Discussion** | **Consensus** | **Discussion** | **Consensus** |  |  |
| **CAT-Pharm**  **Item 12** |  |  |  |  |  |  | Discussed with me how to correctly follow the prescribed therapy | *Changed to*  Explained how to correctly follow the prescribed therapy |  |  |  |  |
| **CAT-Pharm**  **Item 13** | Asked me if I am able to follow the prescribed therapy | *Changed to*  Asked about my ability to follow the prescribed therapy |  |  |  |  |  |  |  |  |  |  |
| **CAT-Pharm**  **Item 11** |  |  |  |  | Discussed about side effects of the prescribed therapy and how to manage them | *Changed to*  Discussed how to manage any side effects of the prescribed therapy |  |  |  |  |  |  |
| **Demographic Question 1** | | Age | *Changed to*  How old are you? |  |  |  |  |  |  |  |  | |
| **Demographic Question 2** | | Gender | *Changed to*  Whati is your gender? |  |  |  |  |  |  |  |  | |
| **Demographic Question 4** | | Nationality | *Changed to*  How would you describe your race or ethnicity? |  |  |  |  |  |  |  |  | |
