# Supplementary material for: Developing and piloting a communication assessment tool assessing patient perspectives on communication with pharmacists (CAT-Pharm)
Source: Int J Clin Pharm. 2022 Feb 24;44(4):1037–45. doi: 10.1007/s11096-022-01382-y (PMC9393125; doi:10.1007/s11096-022-01382-y)
Supplement: Supplementary file 4 — Supplementary file4 (DOC 115 kb) [file 11096_2022_1382_MOESM4_ESM.doc]

**Pharmacist’s Name:**

**Communication Assessment Tool**

Communication with patients is a very important part of health care. We would like to know how you feel about the way your pharmacist communicated with you. **Your answers are completely confidential, so please be as open and honest as you can**.

Your participation is completely voluntary and will not affect your medical treatment in any way.

Please rate the pharmacist’s communication about your prescribed medication therapy. Mark your answer for each item below. Thank you very much.

| **The pharmacist…** | **Poor** | **Fair** | **Good** | **Very Good** | **Excellent** |
| --- | --- | --- | --- | --- | --- |
| 1. Greeted me in a way that made me feel comfortable | 1 | 2 | 3 | 4 | 5 |
| 1. Treated me with respect | 1 | 2 | 3 | 4 | 5 |
| 1. Showed interest in my ideas about the prescribed therapy | 1 | 2 | 3 | 4 | 5 |
| 1. Understood my main health concerns | 1 | 2 | 3 | 4 | 5 |
| 1. Explained how to correctly follow the prescribed therapy | 1 | 2 | 3 | 4 | 5 |
| 1. Let me talk without interruptions | 1 | 2 | 3 | 4 | 5 |
| 1. Gave me as much information as I wanted | 1 | 2 | 3 | 4 | 5 |
| 1. Talked in terms I could understand |  |  |  |  |  |
| 1. Checked to be sure I understood everything | 1 | 2 | 3 | 4 | 5 |
| 1. Encouraged me to ask questions | 1 | 2 | 3 | 4 | 5 |
| 1. Discussed how to manage any side effects of the prescribed therapy | 1 | 2 | 3 | 4 | 5 |
| 1. Discussed next steps, including any follow-up plans | 1 | 2 | 3 | 4 | 5 |
| 1. Asked about my ability to follow the prescribed therapy | 1 | 2 | 3 | 4 | 5 |
| 1. Spent the right amount of time with me | 1 | 2 | 3 | 4 | 5 |
| 1. Discussed possible interactions of the prescribed therapy with other medicines or foods | 1 | 2 | 3 | 4 | 5 |

**~ continues on other side ~**

Copyright © 2004/2016 – Gregory Makoul, PhD – All rights reserved – Non-commercial, educational use permitted

Comments:

**************************************************************************************

This set of questions about the patient is for statistical purposes. Your own responses are completely confidential. Please mark one answer for each question.

1. How old are you? _1_ 24 or younger

2 25-44

3 45-64

4 65-84

5 85 or older

1. What is your gender? _1_ Male

2 Female

3 Other _______________________

1. Have you seen this pharmacist before? _1_ No

2 Yes, but only once

3 Yes, more than once

1. How would you describe _1_ Caucasian

your race or ethnicity? _2_ African

3 African-American

4 Hispanic or Latino

5 Aasian

6 Other _______________________

1. Were you the patient today? _1_ Yes

2 No, I was with the patient today

**Thank you very much.**
