# Supplementary material for: Developing and piloting a communication assessment tool assessing patient perspectives on communication with pharmacists (CAT-Pharm)
Source: Int J Clin Pharm. 2022 Feb 24;44(4):1037–45. doi: 10.1007/s11096-022-01382-y (PMC9393125; doi:10.1007/s11096-022-01382-y)
Supplement: Supplementary file 5 — Supplementary file5 (DOCX 35 kb) [file 11096_2022_1382_MOESM5_ESM.docx]

**Supplementary table 5.1**. **Pearson’s correlation (N* = 247)**

| **CAT-Pharm Items** | | **Item**  **1** | **Item**  **2** | **Item**  **3** | **Item**  **4** | **Item**  **5** | **Item**  **6** | **Item**  **7** | **Item**  **8** | **Item**  **9** | **Item 10** | **Item 11** | **Item 12** | **Item 13** | **Item 14** | **Item 15** |
| --- | --- | --- | --- | --- | --- | --- | --- | --- | --- | --- | --- | --- | --- | --- | --- | --- |
| **Item 1** | **Pearson correlation** | 1 | .861^**^ | .682^**^ | .712^**^ | .637^**^ | .494^**^ | .456^**^ | .453^**^ | .423^**^ | .629^**^ | .642^**^ | .688^**^ | .383^**^ | .319^**^ | .707^**^ |
|  | **Two-tailed significance** |  | 0.000 | 0.000 | 0.000 | 0.000 | 0.000 | 0.000 | 0.000 | 0.000 | 0.000 | 0.000 | 0.000 | 0.000 | 0.000 | 0.000 |
| **Item 2** | **Pearson correlation** | .861^**^ | 1 | .688^**^ | .652^**^ | .558^**^ | .485^**^ | .397^**^ | .385^**^ | .384^**^ | .642^**^ | .678^**^ | .703^**^ | .318^**^ | .293^**^ | .689^**^ |
|  | **Two-tailed significance** | 0.000 |  | 0.000 | 0.000 | 0.000 | 0.000 | 0.000 | 0.000 | 0.000 | 0.000 | 0.000 | 0.000 | 0.000 | 0.000 | 0.000 |
| **Item 3** | **Pearson correlation** | .682^**^ | .688^**^ | 1 | .616^**^ | .630^**^ | .554^**^ | .528^**^ | .497^**^ | .473^**^ | .624^**^ | .563^**^ | .619^**^ | .409^**^ | .422^**^ | .637^**^ |
|  | **Two-tailed significance** | 0.000 | 0.000 |  | 0.000 | 0.000 | 0.000 | 0.000 | 0.000 | 0.000 | 0.000 | 0.000 | 0.000 | 0.000 | 0.000 | 0.000 |
| **Item 4** | **Pearson correlation** | .712^**^ | .652^**^ | .616^**^ | 1 | .574^**^ | .483^**^ | .405^**^ | .373^**^ | .357^**^ | .632^**^ | .613^**^ | .691^**^ | .369^**^ | .259^**^ | .694^**^ |
|  | **Two-tailed significance** | 0.000 | 0.000 | 0.000 |  | 0.000 | 0.000 | 0.000 | 0.000 | 0.000 | 0.000 | 0.000 | 0.000 | 0.000 | 0.000 | 0.000 |
| **Item 5** | **Pearson correlation** | .637^**^ | .558^**^ | .630^**^ | .574^**^ | 1 | .617^**^ | .577^**^ | .543^**^ | .590^**^ | .514^**^ | .499^**^ | .600^**^ | .421^**^ | .460^**^ | .592^**^ |
|  | **Two-tailed significance** | 0.000 | 0.000 | 0.000 | 0.000 |  | 0.000 | 0.000 | 0.000 | 0.000 | 0.000 | 0.000 | 0.000 | 0.000 | 0.000 | 0.000 |
| **Item 6** | **Pearson correlation** | .494^**^ | .485^**^ | .554^**^ | .483^**^ | .617^**^ | 1 | .672^**^ | .558^**^ | .642^**^ | .678^**^ | .552^**^ | .545^**^ | .491^**^ | .534^**^ | .526^**^ |
|  | **Two-tailed significance** | 0.000 | 0.000 | 0.000 | 0.000 | 0.000 |  | 0.000 | 0.000 | 0.000 | 0.000 | 0.000 | 0.000 | 0.000 | 0.000 | 0.000 |
| **Item 7** | **Pearson correlation** | .456^**^ | .397^**^ | .528^**^ | .405^**^ | .577^**^ | .672^**^ | 1 | .680^**^ | .668^**^ | .511^**^ | .441^**^ | .525^**^ | .542^**^ | .529^**^ | .436^**^ |
|  | **Two-tailed significance** | 0.000 | 0.000 | 0.000 | 0.000 | 0.000 | 0.000 |  | 0.000 | 0.000 | 0.000 | 0.000 | 0.000 | 0.000 | 0.000 | 0.000 |
| **Item 8** | **Pearson correlation** | .453^**^ | .385^**^ | .497^**^ | .373^**^ | .543^**^ | .558^**^ | .680^**^ | 1 | .781^**^ | .583^**^ | .450^**^ | .475^**^ | .557^**^ | .574^**^ | .455^**^ |
|  | **Two-tailed significance** | 0.000 | 0.000 | 0.000 | 0.000 | 0.000 | 0.000 | 0.000 |  | 0.000 | 0.000 | 0.000 | 0.000 | 0.000 | 0.000 | 0.000 |
| **Item 9** | **Pearson correlation** | .423^**^ | .384^**^ | .473^**^ | .357^**^ | .590^**^ | .642^**^ | .668^**^ | .781^**^ | 1 | .570^**^ | .404^**^ | .432^**^ | .613^**^ | .705^**^ | .399^**^ |
|  | **Two-tailed significance** | 0.000 | 0.000 | 0.000 | 0.000 | 0.000 | 0.000 | 0.000 | 0.000 |  | 0.000 | 0.000 | 0.000 | 0.000 | 0.000 | 0.000 |
| **Item 10** | **Pearson correlation** | .629^**^ | .642^**^ | .624^**^ | .632^**^ | .514^**^ | .678^**^ | .511^**^ | .583^**^ | .570^**^ | 1 | .736^**^ | .650^**^ | .472^**^ | .437^**^ | .678^**^ |
|  | **Two-tailed significance** | 0.000 | 0.000 | 0.000 | 0.000 | 0.000 | 0.000 | 0.000 | 0.000 | 0.000 |  | 0.000 | 0.000 | 0.000 | 0.000 | 0.000 |
| **Item 11** | **Pearson correlation** | .642^**^ | .678^**^ | .563^**^ | .613^**^ | .499^**^ | .552^**^ | .441^**^ | .450^**^ | .404^**^ | .736^**^ | 1 | .749^**^ | .461^**^ | .370^**^ | .715^**^ |
|  | **Two-tailed significance** | 0.000 | 0.000 | 0.000 | 0.000 | 0.000 | 0.000 | 0.000 | 0.000 | 0.000 | 0.000 |  | 0.000 | 0.000 | 0.000 | 0.000 |
| **Item 12** | **Pearson correlation** | .688^**^ | .703^**^ | .619^**^ | .691^**^ | .600^**^ | .545^**^ | .525^**^ | .475^**^ | .432^**^ | .650^**^ | .749^**^ | 1 | .507^**^ | .369^**^ | .780^**^ |
|  | **Two-tailed significance** | 0.000 | 0.000 | 0.000 | 0.000 | 0.000 | 0.000 | 0.000 | 0.000 | 0.000 | 0.000 | 0.000 |  | 0.000 | 0.000 | 0.000 |
| **Item 13** | **Pearson correlation** | .383^**^ | .318^**^ | .409^**^ | .369^**^ | .421^**^ | .491^**^ | .542^**^ | .557^**^ | .613^**^ | .472^**^ | .461^**^ | .507^**^ | 1 | .673^**^ | .452^**^ |
|  | **Two-tailed significance** | 0.000 | 0.000 | 0.000 | 0.000 | 0.000 | 0.000 | 0.000 | 0.000 | 0.000 | 0.000 | 0.000 | 0.000 |  | 0.000 | 0.000 |
| **Item 14** | **Pearson correlation** | .319^**^ | .293^**^ | .422^**^ | .259^**^ | .460^**^ | .534^**^ | .529^**^ | .574^**^ | .705^**^ | .437^**^ | .370^**^ | .369^**^ | .673^**^ | 1 | .361^**^ |
|  | **Two-tailed significance** | 0.000 | 0.000 | 0.000 | 0.000 | 0.000 | 0.000 | 0.000 | 0.000 | 0.000 | 0.000 | 0.000 | 0.000 | 0.000 |  | 0.000 |
| **Item 15** | **Pearson correlation** | .707^**^ | .689^**^ | .637^**^ | .694^**^ | .592^**^ | .526^**^ | .436^**^ | .455^**^ | .399^**^ | .678^**^ | .715^**^ | .780^**^ | .452^**^ | .361^**^ | 1 |
|  | **Two-tailed significance** | 0.000 | 0.000 | 0.000 | 0.000 | 0.000 | 0.000 | 0.000 | 0.000 | 0.000 | 0.000 | 0.000 | 0.000 | 0.000 | 0.000 |  |
| ***Total sample interviewed**  **** Significant correlation** | | | | | | | | | | | | | | | | |

**Supplementary table 5.2**. **Confirmatory factor analysis (N* = 247)**

| **CAT-Pharm Items** | | **Factor 1** | **Factor 2** |
| --- | --- | --- | --- |
| **Item 1** | Greeted me in a way that made me feel comfortable | **0.904** | -0.044 |
| **Item 2** | Treated me with respect | **0.946** | -0.123 |
| **Item 3** | Showed interest in my ideas about the prescribed therapy | **0.677** | 0.193 |
| **Item 4** | Understood my main health concerns | **0.909** | -0.125 |
| **Item 5** | Explained how to correctly follow the prescribed therapy | **0.484** | 0.388 |
| **Item 6** | Let me talk without interruptions | **0.304** | 0.59 |
| **Item 7** | Gave me as much information as I wanted | 0.132 | **0.741** |
| **Item 8** | Talked in terms I could understand | 0.064 | **0.803** |
| **Item 9** | Checked to be sure I understood everything | -0.04 | **0.925** |
| **Item 10** | Encouraged me to ask questions | 0.667 | **0.258** |
| **Item 11** | Discussed how to manage any side effects of the prescribed therapy | 0.781 | **0.067** |
| **Item 12** | Discussed next steps, including any follow-up plans | 0.816 | **0.084** |
| **Item 13** | Asked about my ability to follow the prescribed therapy | 0.035 | **0.755** |
| **Item 14** | Spent the right amount of time with me | -0.142 | **0.904** |
| **Item 15** | Discussed possible interactions of the prescribed therapy with other medicines or foods | 0.861 | **0.01** |
| ***Explained Variance (%)*** | | *57.318* | *12.726* |
| ***Cumulative Variance (%)*** | | *57.318* | *70.044* |
| ***KMO*** | | *0.921* | |
| ***χ2 (df)*** | | *2969.339 (105)* | |
| ***p-value*** | | *<0.01* | |

*Totale sample interviewed

Abbreviations: df, Degrees of freedom; KMO, Kaiser-Meyer-Olkin.

Notes: Extraction method: Principal component analysis; Rotation method: Oblimin with Kaiser normalisation.
